# *Echinococcus granulosus *sensu stricto and antigen B may decrease inflammatory bowel disease through regulation of M1/2 polarization

**DOI:** 10.1186/s13071-022-05498-y

**Published:** 2022-10-27

**Authors:** Jianling Bao, Wenjing Qi, Chang Sun, Mengxiao Tian, Hongjie Jiao, Gang Guo, Baoping Guo, Yuan Ren, Huajun Zheng, Yuezhu Wang, Mei Yan, Zhaoxia Zhang, Donald P. McManus, Jun Li, Wenbao Zhang

**Affiliations:** 1 State Key Laboratory of Pathogenesis, Prevention and Treatment of High Incidence Diseases in Central Asia, The First Affiliated Hospital of Xinjiang Medical University, Xinjiang Medical University, Urumqi, 830054 Xinjiang China; 2 Basic Medicine College, Xinjiang Medical University, Urumqi, 830011 Xinjiang China; 3 NHC Key Laboratory of Reproduction Regulation, Shanghai Institute of Planned Parenthood Research, Fudan University, Shanghai, 200032 China; 4 Shanghai-MOST Key Laboratory of Health and Disease Genomics, Chinese National Human Genome Center at Shanghai, Shanghai, 201203 China; 5 Molecular Parasitology Laboratory, Infectious Diseases Program, QIMR Berghofer Medical Research Institute, Brisbane, QLD Australia

**Keywords:** *Echinococcus granulosus*, Macrophage polarization, Inflammatory bowel disease (IBD), Antigen B

## Abstract

**Background:**

Inflammatory bowel disease (IBD) is a chronic idiopathic disease characterized by inflammation-related epithelial barrier damage in the intestinal tract. Helminth infection reduces autoimmune disease symptoms through regulation of inflammatory responses based on hygiene theory. However, the underlying mechanisms remain unclear.

**Methods:**

BALB/c mice were infected with microcysts of *E. granulosus* sensu stricto and drank water containing 3.5% dextran sodium sulfate (DSS) at the 100th day post-infection. After 7 days of drinking DSS, the mouse body weight change and disease activity index (DAI) were recorded every day, and colon length and histological score were evaluated after sacrifice. After injection with antigen B (AgB), inducible nitric oxide synthase (iNOS) and Fizz1 expression and F4/80^+^CD11c^+^ M1 and F4/80^+^CD206^+^ M2 in the peritoneal cells and colon tissues were analysed by qPCR and flow cytometry, respectively. Gut microbiota were profiled by 16S rRNA sequencing of the mouse faecal samples. For in vitro assay, RAW264.7 macrophages were cultured in medium containing AgB before induction by lipopolysaccharide (LPS). Then, NO in the supernatant was measured, and the expression of cytokine genes associated with macrophages were determined by qRT-PCR.

**Results:**

*Echinococcus granulosus* s.s. infection and AgB significantly reduced the symptoms and histological scores of IBD induced by DSS (*P* < 0.05). Flow cytometry showed that AgB inoculation increased F4/80^+^ and CD206^+^ in peritoneal cells. The results of qPCR showed that AgB significantly decreased iNOS and increased Fizz1 expression in the colon of mice inoculated by DSS (*P* < 0.05). Furthermore, AgB injection led to significant changes in the profiles of five genera (*Paraprevotella*, *Odoribacter*, *Clostridium* cluster XlVa, *Oscillibacter*, and *Flavonifractor*) in faecal samples. In vitro analysis showed that AgB reduced NO levels (*P* < 0.01), with a significant decrease in iNOS expression (*P* < 0.05) in RAW264.7 cells induced by LPS.

**Conclusions:**

*Echinococcus granulosus* infection and AgB may improve IBD conditions by inducing an M2-predominant cellular (F4/80^+^ CD206^+^) profile and decreasing type 1 macrophages (F4/80^+^CD11c^+^) in the intestinal lamina propria. In addition, AgB intervention induced changes in the microbiota condition of the gastrointestinal duct and reversed NO expression. Thus, AgB may be a drug candidate for IBD treatment.

**Graphical Abstract:**

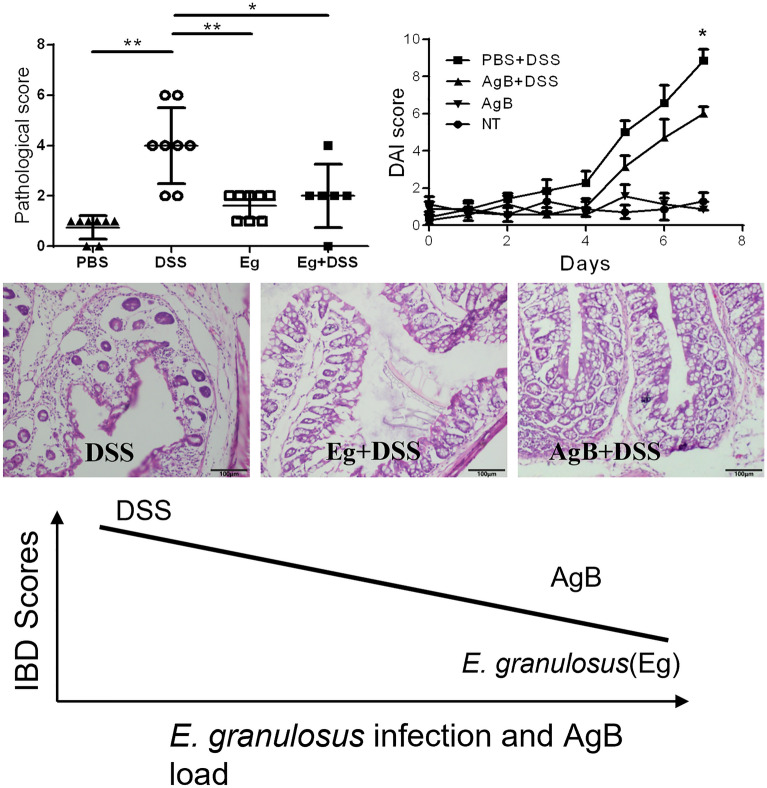

**Supplementary Information:**

The online version contains supplementary material available at 10.1186/s13071-022-05498-y.

## Background

*Echinococcus granulosus* sensu stricto is a tapeworm that causes zoonotic cystic echinococcosis (CE) [1, 2]. This disease has a cosmopolitan distribution, with high endemicity in Central Asia (including western China), Africa, Eastern Europe, and South America [3]. Herbivore intermediate hosts and humans are infected by swallowing echinococcal eggs released from the adult worms of *E. granulosus* in the dog’s intestine. Oncospheres hatch from eggs and are activated in the small intestine of the intermediate host or humans, and settle in the internal organs, mostly the liver and lungs; they then grow slowly to cysts containing echinococcal cyst fluid (ECF) and protoscoleces (PSCs) [4]. The life-cycle of this tapeworm is completed when a dog eats the offal of intermediate animals containing cysts with PSCs, which develop into adult worms in the intestine of the definitive dog host.

Inflammatory bowel diseases (IBDs), including Crohn’s disease (CD) and ulcerative colitis (UC), are characterized by exacerbated chronic inflammation of the gastrointestinal tract [5]. The underlying mechanism causing IBD is not clear. Some studies have shown that IBD is associated with dysregulated immune responses [6].

Macrophages are heterogeneous immune cells that can alter their phenotype and physiology in response to environmental cues, and these cells are classified into M1 (classic) macrophages or M2 (alternative) macrophages. Macrophages play an important role in maintaining intestinal homeostasis via their ability to orchestrate responses to the normal microbiota as well as pathogens. Lamina propria M1 macrophages invading intestinal tissues contribute directly to disrupting the epithelial barrier through deregulation of tight junction proteins and induction of epithelial cell apoptosis, thus driving intestinal inflammation in IBD. These M1 macrophages shift the balance in the local macrophage compartment towards a proinflammatory state [7].

The hygiene hypothesis may explain the higher incidence of IBD in developed countries than in developing countries, likely due to the difference in exposure to microbes and parasites [8]. Recent studies have demonstrated that parasitic infections may show promise in the treatment of some autoimmune disorders, including IBD and multiple sclerosis [9, 10]. We previously reported that *E. granulosus* s.s. infection alleviated asthma symptoms by enhancing interleukin 10 (IL-10) and downregulating IL-5 and IL-17A [11]. However, whether major components of *E. granulosus* s.s. ECF, such as antigen B (AgB), play a role in modulating these autoimmune disorders is unknown.

In this study, we showed that *E. granulosus* s.s. infection [12] and AgB significantly reduced IBD responses induced by dextran sodium sulfate (DSS), possibly through the impact on M1/M2 polarization in the gastrointestinal tissues.

## Methods

### Experimental design

**Fig. 1:**
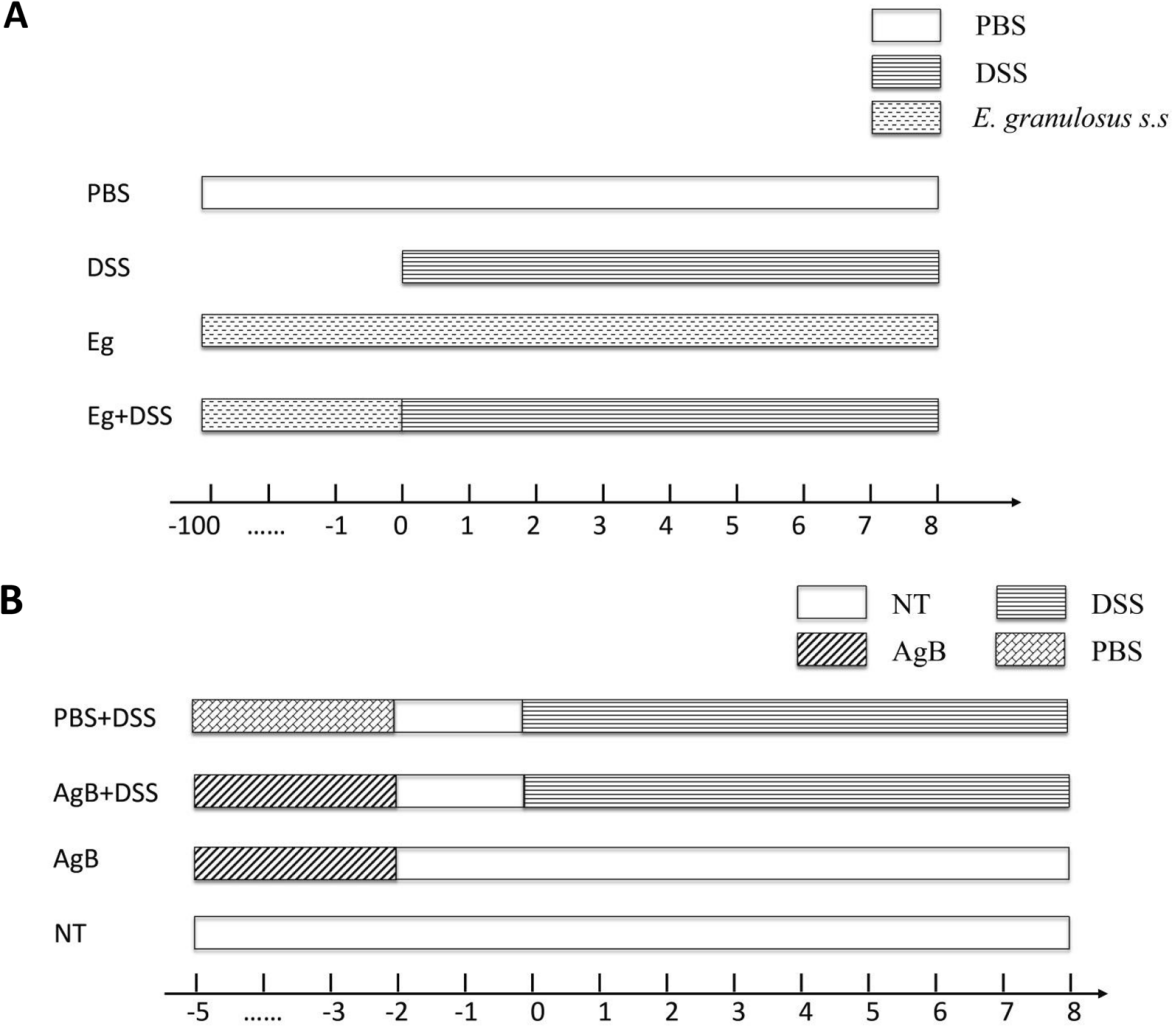
Mice were randomly divided into four experimental groups (*n* = 10 per group). **A** Experimental plan for testing *E. granulosus* s.s. infection and IBD. **B** Experimental plan for determining the effect of AgB against IBD

To determine whether *E. granulosus* s.s. infection induces a protective effect against IBD, BALB/c mice were randomly divided into four groups (*n* = 6–10) as shown in Fig. 1A. The PBS (phosphate-buffered saline) group mice (PBS) were each injected intraperitoneally (i.p.) with 1.0 ml PBS; DSS group mice (DSS) received 3.5% (w/v) of DSS in their drinking water for 7 days to induce acute colitis. *Echinococcus granulosus* s.s. infection group mice (Eg) each received i.p. transplantation of 35 *E. granulosus* s.s. microcysts, and the *E. granulosus* s.s. infection + DSS group (Eg+DSS) mice were given drinking water containing 3.5% DSS for 7 days beginning day 100 after *E. granulosus* s.s. infection.

To determine whether *E. granulosus* AgB protects mice against IBD, four groups of mice were designated (Fig. 1B). PBS+DSS group mice (PBS+DSS) were each injected i.p. with 1.0 ml/day PBS for 5 days, allowed to rest for 2 days, then concomitantly injected i.p. with 3.5% of DSS for 7 days. In the AgB+DSS group (AgB+DSS), each mouse was injected with 100 μg/day AgB for 5 days, allowed to rest for 2 days, and then drank water containing 3.5% of DSS (w/v) for 7 days. AgB group mice (AgB) were each injected with 100 μg AgB for 5 days, and a no-treatment group (NT) served as the negative control group.

### Preparation of cultured *E. granulosus* s.s. cysts and animal infection

In order to prepare microcysts of the tapeworm for infection of mice, fresh *E. granulosus* s.s. PSCs were aspirated from *E. granulosus* s.s. cysts from sheep livers collected from a slaughterhouse in Urumqi, Xinjiang Uyghur Autonomous Region, People’s Republic of China. The PSCs were digested with 1% (w/v) pepsin and cultured to obtain microcysts using a published procedure [12]. Pathogen-free female BALB/c mice, aged 6 weeks, were purchased from Beijing Vital River Laboratory Animal Technology Co., Ltd. All animals were housed at the animal facility of the First Affiliated Hospital of Xinjiang Medical University (FAH-XMU). *Echinococcus granulosus* s.s. infection groups of mice, including animals receiving i.p. transplantation of 35 *E. granulosus* s.s. microcysts (diameter, 200–300 μm) in 0.4 ml Roswell Park Memorial Institute (RPMI) 1640 medium using a 1.0-ml syringe, and control mice, were each injected with 1.0 ml RPMI 1640 medium. After 90 days of microcyst transplantation, an enzyme-linked immunoassay (ELISA) plate coated with ECF was used to confirm the success of infection, and positive animals were assigned to the Eg and Eg+DSS groups, with 8–10 mice in each group. Mice with ELISA-negative results were excluded.

### Preparation of ECF, AgB, and intervention

AgB was purified from *E. granulosus* s.s. ECF according to a previously published method [13]. In brief, after collection from echinococcal cysts, ECF was centrifuged at 10,000×*g* for 20 min at 4 °C, and the supernatant was filtered through 0.45-μm filter membranes (Millipore). The clarified ECF was used for i.p. injection of mice. To purify AgB, ECF was precipitated by adding sodium acetate at a final concentration of 5 mM, pH 5.0. After centrifugation at 5000×*g* for 15 min at 4 °C, the sediment was resuspended in PBS and then heated in boiling water for 5 min. After further centrifugation at 10,000×*g* for 15 min at 4 °C, the supernatant was collected and filtered through a 0.22-μm filter. Endotoxin was removed using endotoxin removal resin (ToxinEraser™ Endotoxin Removal Kit, GenScript Biotech Corp., China). The absence of lipopolysaccharide (LPS) in the supernatant was further verified using gel clot assays (ToxinSensor™ Gel Clot Endotoxin Assay Kit, GenScript Biotech Corp., China). Mice in the AgB-treated group received purified native AgB daily (100 μg/day) by i.p. injection for 5 days.

### Murine model of DSS-induced acute colitis

A BALB/c IBD model was established according to previously reported methods [14]. DSS (36–50 kDa, MP Biomedicals) was prepared at a concentration of 3.5% (w/v) and was used as drinking water for IBD experimental mice. The DSS drinking water bottle was changed every 2 days. Control mice received autoclaved water only. A successful IBD model induced by DSS was characterized by constant weight loss, development of diarrhoea, and blood in the faeces after the third day of DSS administration. The mice were finally sacrificed on day 8 of DSS administration.

### Disease activity index (DAI) assessment

The DAI score was calculated each day and used to evaluate the grade and extent of intestinal inflammation in the mice along with body weight change, stool consistency, and blood in the stool. Weight loss was scored as follows: 0 = no change; 1 = 1–5%; 2 = 5–10%; 3 = 10–20%; 4 = > 20%. Stool consistency was scored in three grades: 0 = normal well-formed faecal pellets; 2 = loose, pasty coloured, and semi-formed stools that did not adhere to the anus; and 4 = diarrhoea characterized by liquid stools that adhered to the anus. Blood in the stool was scored as follows: 0 = normal; 2 = slight bleeding, and 4 = gross bleeding. The DAI was calculated as the sum of the individual scores for bloody stool, stool consistency, and body weight loss, as described previously [15].

### Colon length and histopathology

After euthanasia of the mice by CO_2_, the colon was isolated and carefully cleaned, and the length measured. For histology, 1 cm of the distal colon was fixed in 4% (v/v) formalin, dehydrated in 30% (w/v) sucrose, mounted in optimal cutting temperature (OCT) blocks, and then cut into 4-μm-thick frozen sections. The sections were stained with haematoxylin and eosin (H&E) to study histological differences between groups or were stained with antibodies for immunohistochemical analysis.

Histological evaluation was performed by three investigators blinded to the animal groups, and inflammation was graded as follows: severity of inflammation (0–3, representing none, slight, moderate, and severe, respectively), extent of injury (0–3, representing none, mucosal, mucosal, submucosal, and transmural, respectively), and crypt damage (0–4, representing none, basal 1/3 damaged, basal 2/3 damaged, only surface epithelium intact, and entire crypt/epithelium lost, respectively). The histological score ranged from 0 to 10.

### Acquisition of abdominal lavage fluid

Peritoneal lavage fluid was collected from these euthanized mice, and 8 ml iced PBS was quickly and forcefully injected into the abdominal cavity. Then, 5 ml turbid liquid was aspirated after 3–5 min.

### Isolation of lamina propria mononuclear cells (LPMCs)

LPMCs were collected by dissection from mouse large intestines for flow cytometry analysis. In brief, each dissected colon was opened longitudinally, transferred into a centrifuge tube containing RPMI 1640/5 mM ethylenediaminetetraacetic acid (EDTA), and incubated and rotated at 200 rpm/min for 30 min at 37 °C. The colon tissue was then minced, transferred into a new tube with 2 ml collagenase/DNase solution (100 ml of RPMI 1640 containing collagenase type IV, 0.1 g; DNase I, 0.004 g), and incubated for 40 min at 37 °C. The resulting cell suspension was passed through a 70-μm cell sieve and centrifuged at 1700 rpm/min for 10 min. The cell pellet was resuspended in 6 ml of 40% (v/v) Percoll solution and overlaid on top of 3 ml of 75% (v/v) Percoll. The cells in the intermediate layer were collected after centrifugation at 2000 rpm/min for 20 min. The collected cells were again separated using 75% Percoll and aspirated and resuspended in RPMI 1640 containing 10% (v/v) foetal bovine serum (FBS) for flow cytometry. Colons with a length of 1 cm were cut off and frozen in an −80 °C refrigerator for quantitative reverse transcriptase polymerase chain reaction (qRT-PCR) analysis.

### Flow cytometry

Aliquots of 10^5^ cells/30 μl of staining buffer per well were incubated with anti-CD16/CD32 antibodies for 15 min in the dark to block non-specific binding of antibodies to the Fc receptors. Subsequently, these cells were respectively stained with the following surface markers for 15 min with 1 μg of primary antibodies: allophycocyanin (APC)-Cy7-labelled anti-CD45, phycoerythrin (PE)-labelled anti-F4/80, fluorescein isothiocyanate (FITC)-labelled anti-CD11c, APC-labelled anti-CD206. Corresponding fluorochrome-labelled isotype control antibodies were used for staining controls. All antibodies were purchased from BD Biosciences (San Jose, CA, USA). All cells were subsequently detected using a FACSCalibur Flow Cytometer (BD Biosciences, San Jose, CA, USA), and analysed using FlowJo software (Tree Star, Inc., San Carlos, CA, USA).

### RAW264.7 cell culture

The RAW264.7 macrophage cell line (ATCC, USA) was purchased from the Shanghai Institutes of Biochemistry and Cell Biology and cultured in RPMI 1640 medium supplemented with 10% (v/v) FBS and penicillin (50 U/ml)/streptomycin (50 μg/ml) in a 5% CO_2_ atmosphere at 37 °C. Prior to seeding, cells with 70–80% confluence were harvested once every 2 days. For the macrophage differentiation studies, approximately 1.0 × 10^5^ cells were seeded into 12-well plates (Costar) and incubated as described above. After 4 h of incubation, the cells were stimulated with LPS (100 ng/ml) and interferon gamma (IFN-γ) (25 ng/ml) or IL-4 (100 ng/ml) and AgB protein (500 ng/ml). The cell morphology was then observed by a microscope after 12 h stimulation. The remainder of the cells were collected and frozen in the −80 °C refrigerator for testing NO, which was tested by the nitrate reductase method (NO assay kit, Nanjing Jiancheng Bioengineering Institute, China).

### Cytokine gene expression analyses by qRT-PCR

Total RNA was extracted from about 1 mg of colon tissue using TRIzol reagent (15596-026, Invitrogen, China) and converted into complementary DNA (cDNA) using a PrimeScript RT reagent kit with the gDNA [genomic DNA] Eraser (RR047A, TaKaRa Bio, Inc., Dalian, China). The reaction conditions for qRT-PCR amplification were as follows: 94 °C for 3 min, followed by 35 cycles of 94 °C for 30 s, 60 °C for 30 s, and 72 °C for 30 s. Inducible nitric oxide synthase (iNOS) and Fizz1 messenger RNA (mRNA) levels were quantified relative to the mRNA level of housekeeping gene β-actin. β-actin primers: forward primer 5′-AACTCCATCATGAAGTGTGA-3′ and reverse primer 5′-ACTCCTGCTTGCTGATCCAC-3′. iNOS primers: forward primer 5′-CCCTTCCGAAGTTTCTGGCAGCAGC-3′ and reverse primer 5′-GGCTGTCAGAGCCTCRTGGCTTTGG-3′. Fizz1 primers: forward primer 5′-GGTCCCAGCATATGGATGAGACCATAGA-3′ and reverse primer 5′-CACCTCTTCACTCGAGGGACAGTTGGCAGC-3′.

### PCR amplification and 16S ribosomal RNA (rRNA) gene sequencing

The faecal DNA of mice was extracted using the QIAamp DNA Stool Mini Kit (QIAGEN, Hilden, Germany). The V3–V4 hypervariable region of the 16S rRNA gene was amplified using primers 338F and 806R with TransStart FastPfu DNA Polymerase (TransGen, Beijing, China) in 20 cycles. All amplicons were purified using the QIAquick PCR Purification Kit (Qiagen) and pooled with equal concentrations. Then the pooled amplicons were sequenced on an Illumina MiSeq instrument with 2 × 300 bp paired-end sequencing.

### Statistical analysis

Gastrointestinal microbes were assessed using analysis of molecular variance (AMOVA). The input file (biom file) of PICRUSt was calculated using the Mothur software commands “classify.otu” and “make.biom”, and then the input file was uploaded to the online PICRUSt for functional analysis. Differences were determined using STAMP [16].

The R statistical analysis program was used to calculate the coefficient relationship between bacterial genera present and the results of fluorescence-activated cell sorting (FACS) and PCR, using the nonparametric Spearman rank correlation algorithm. A coefficient of > 0.3 or < −0.3 was considered to represent a correlation [17].

Data were analysed using GraphPad Prism 5 software (GraphPad Software, Inc., USA). Statistical differences between groups were determined by one-way analysis of variance (ANOVA) followed by Tukey’s test. Statistical significance was accepted for values of *P* < 0.05.

## Results

### *Echinococcus granulosus* s.s. infection ameliorated the clinical symptoms of DSS-induced colitis

**Fig. 2:**
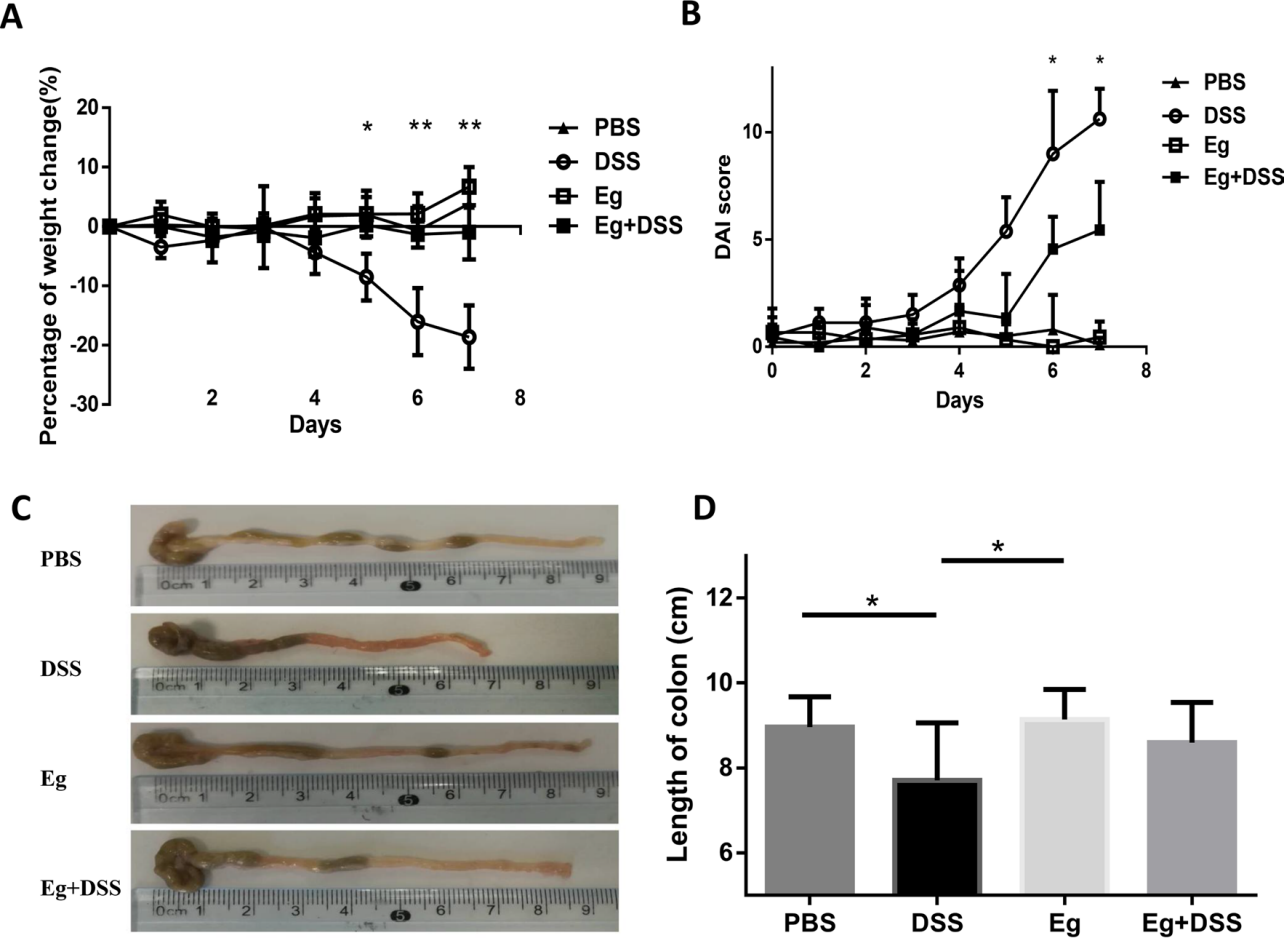
Infection with *E. granulosus* s.s. ameliorated the clinical symptoms of DSS-induced colitis. Course of ulcerative colitis in *E. granulosus* s.s.-infected and uninfected mice following 7 days of DSS treatment. **A** Body weight change. Data represent the mean ± SD (*n* = 7–8 mice in each group). **B** DAI score (*n* = 8–10, mean ± SD). DAI score is used as an index of inflammation in the DSS-induced colitis model. In the DSS-alone group, the DAI score was significantly higher (10.63 ± 1.41, *P* < 0.05) than in the Eg+DSS group (5.45 ± 2.24). **C** Photograph of gross pathology of colons from different groups of mice. **D** Colon lengths from mice infected and uninfected with DSS-induced colitis (*n* = 7–9). Bars represent the mean ± SD for six or more mice per group. These experiments were repeated twice independently (∗ *P* < 0.05, ∗ ∗ *P* < 0.01), and data were analysed by one-way ANOVA and unpaired *t*-test

To determine whether *E. granulosus* s.s. infection impacts IBD progression, mice were infected with 35 microcysts cultured in vitro [12]. At 100 days post-infection, the mice infected with *E. granulosus* s.s. produced relatively high levels of antibodies against ECF antigens, indicating that these mice were successfully infected. The mice harboured 8 to 37 cysts (mean, 16 cysts) at necropsy. The mice given drinking water containing 3.5% DSS suffered body weight loss commencing at day 4 post-DSS administration. At day 7 post-treatment with DSS, the average body weight in the DSS group was reduced by 18.59 ± 5.33% (*P* < 0.05) compared with the average body weight of mice given PBS, which only increased by 3.84 ± 3.78% (Fig. 2A).The weight of mice of the Eg+DSS group was reduced by 4.79 ± 6.67% (*P* < 0.05) compared with that in the PBS group, which was increased by 16.79 ± 8.87% (*P* < 0.05) compared with the average body weight of mice in the DSS group (Fig. 2A). Furthermore, the DAI score indicated improved clinical signs in the Eg+DSS group (*P* < 0.05) including less diarrhoea, fewer gross rectal effects, and no moribund symptoms (Fig. 2B). In addition, the colon length in the DSS group was much shorter (1.248 ± 0.495, *P* < 0.05) than the colon length in the PBS group (Fig. 2C, D). No significant difference was observed between the Eg, Eg+DSS, and PBS groups (Fig. 2C, D).

**Fig. 3:**
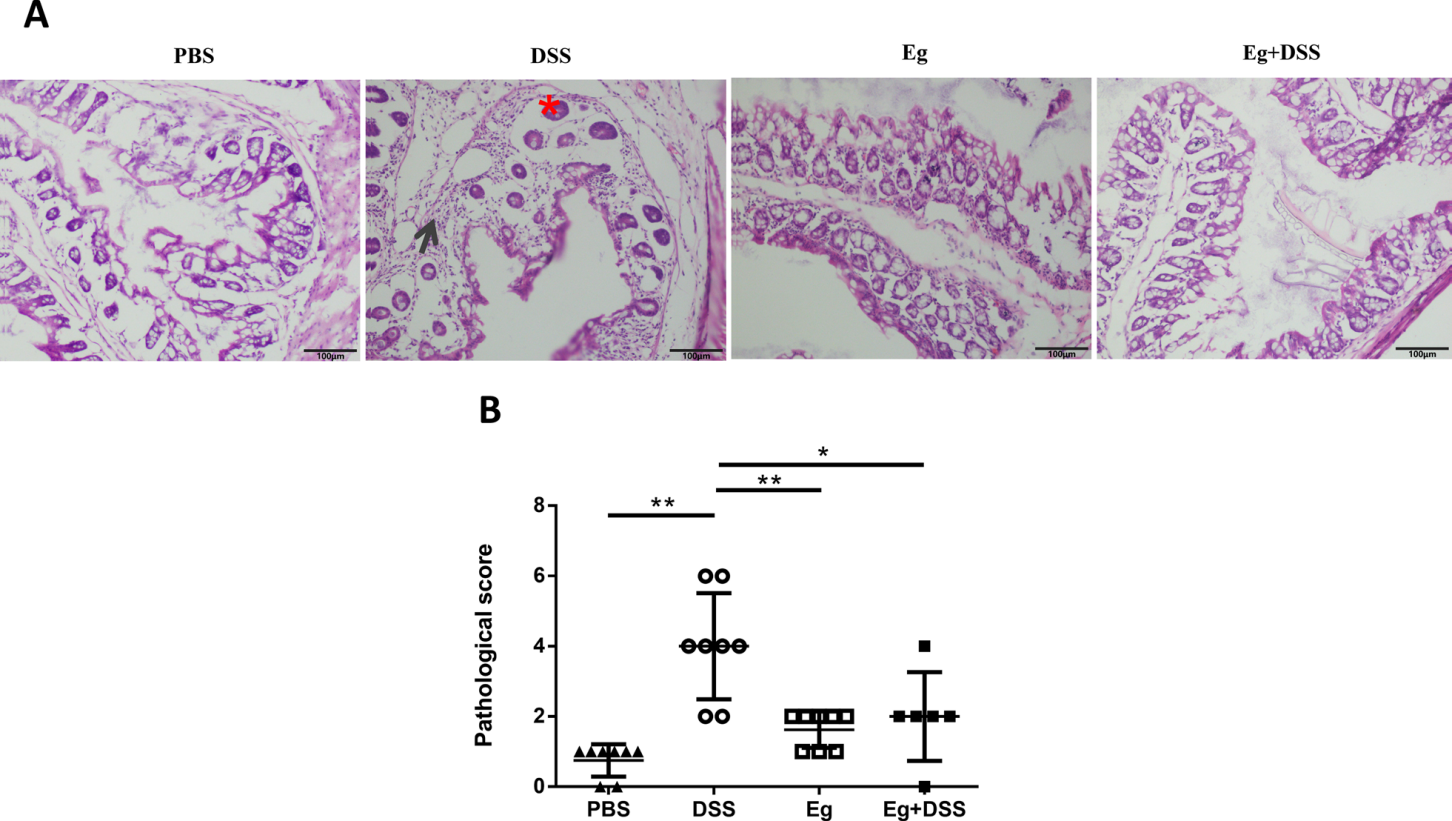
Infection with *E. granulosus* s.s. reduced the pathological score of the colon shown by H&E staining (×200). **A** Colon tissue histology shown by H&E staining indicating colonic inflammation in the different groups: red star, inflammatory cells infiltrated in the mucosa; dark arrow: crypt abscess. **B** Comparison of histological scores for colon cellular infiltration and tissue alterations between the DSS and Eg+DSS groups. In the Eg+DSS group, the histological score was significantly decreased (2 ± 0.52, *P* < 0.05) compared with the DSS group (4 ± 0.53). Unpaired *t*-test was used in data analysis (6–8 mice per group, ∗ *P* < 0.05, ∗ ∗ *P* < 0.01.). These experiments were repeated twice independently

Histological analysis showed that the distal colon had more intense inflammatory cell infiltration extending throughout the mucosa and submucosa, with crypt destruction, complete depletion of goblet cells, and mucosa erosion in the DSS group mice. Histological scores showed that the pathological score was significantly decreased in the Eg, Eg+DSS, and PBS groups compared with the DSS group (*P* < 0.01) (Fig. 3A, B).

### AgB decreased the clinical symptoms of DSS-induced colitis

**Fig. 4:**
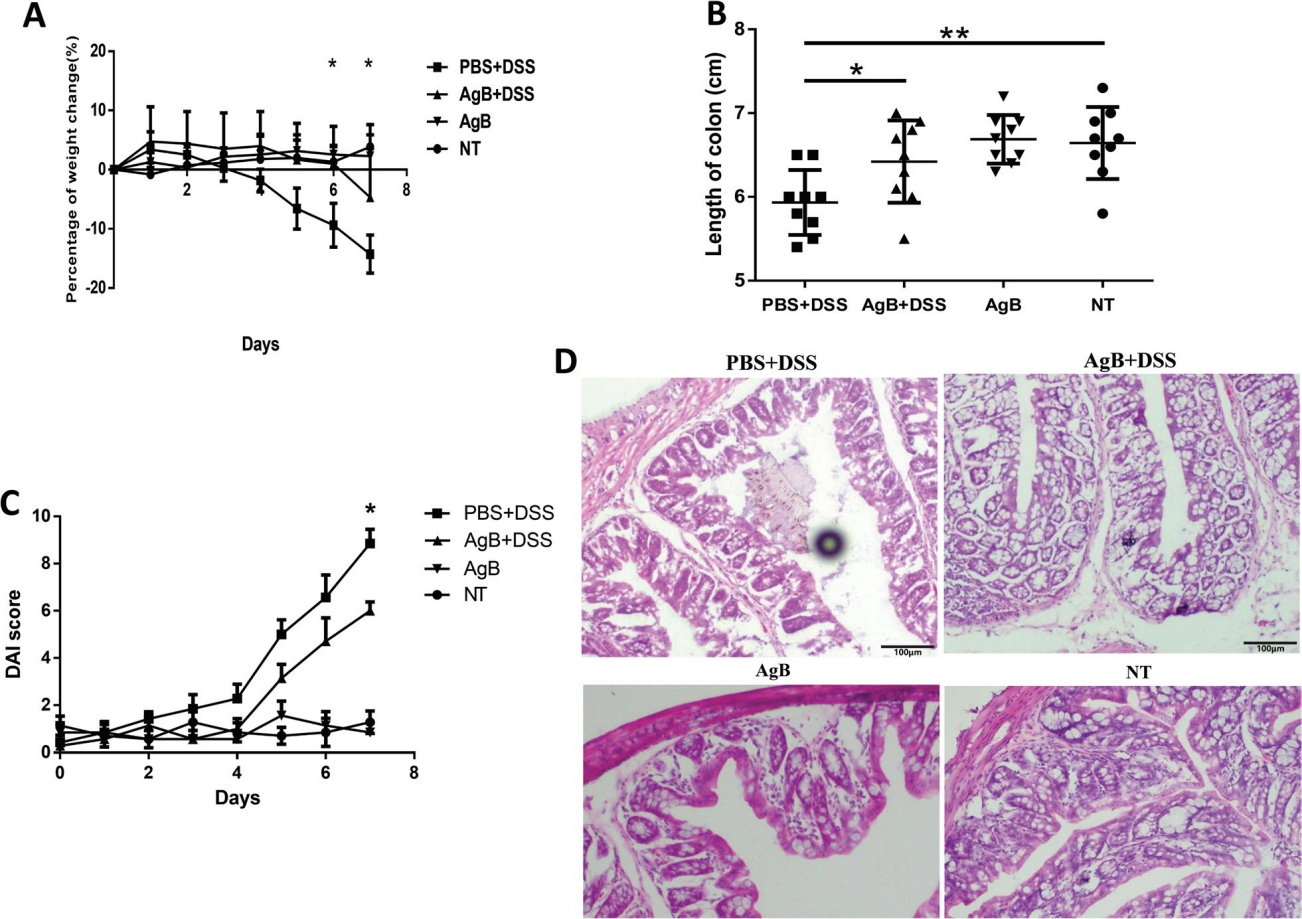
Colonic inflammation was attenuated in the AgB-treated/DSS-induced colitis model. Purified AgB was injected into mice intraperitoneally before DSS administration. The differences between the DSS group and AgB+DSS group are shown: **A** Body weight. Bars represent the mean ± SD for 7–10 mice per group, at day 7 post-treatment with DSS. The average body weight of the PBS+DSS group was reduced by 14.27 ± 3.21% (*P* < 0.05) compared with a reduction in the AgB+DSS group of 4.68 ± 8.24% (*P* < 0.05). **B** Length of colon. Bars represent the mean ± SD for nine mice per group. In the PBS+DSS group, the length of the colon was significantly shortened (5.93 ± 0.13 cm, *P* < 0.05) compared with the AgB+DSS group (6.42 ± 0.16 cm). **C** DAI scores. In the PBS+DSS group, the DAI score was significantly increased (8.86 ± 1.575, *P* < 0.05) compared with the AgB+DSS group (6 ± 1). Bars represent the mean ± SEM for seven mice per group. **D** Colon tissue histology stained with H&E (8–10 mice per group, and the experiment was repeated independently) (×200)

AgB is the major source of secreted protein of *E. granulosus* s.s. in ECF. We purified AgB from ECF and injected each mouse with 100 μg/day for 5 days. PBS+DSS challenge resulted in significant body weight loss (Fig. 4A), colon shortening, and increased DAI score compared with those in the NT group. In the PBS+DSS control mice, the length of the colon was significantly shortened (5.9 ± 0.29 cm) compared with the colon length of the NT group (6.64 ± 0.13 cm, *P* < 0.005), whereas the colon length (6.4 ± 0.16 cm) of the AgB+DSS group was longer than the colon length of the PBS+DSS group (*P* < 0.05, Fig. 4B). No significant difference in colon length was observed between the AgB and NT groups. The DAI scores were significantly lower in the AgB+DSS group than in the PBS+DSS group (*P* < 0.05) (Fig. 4C, D).

**Fig. 5:**
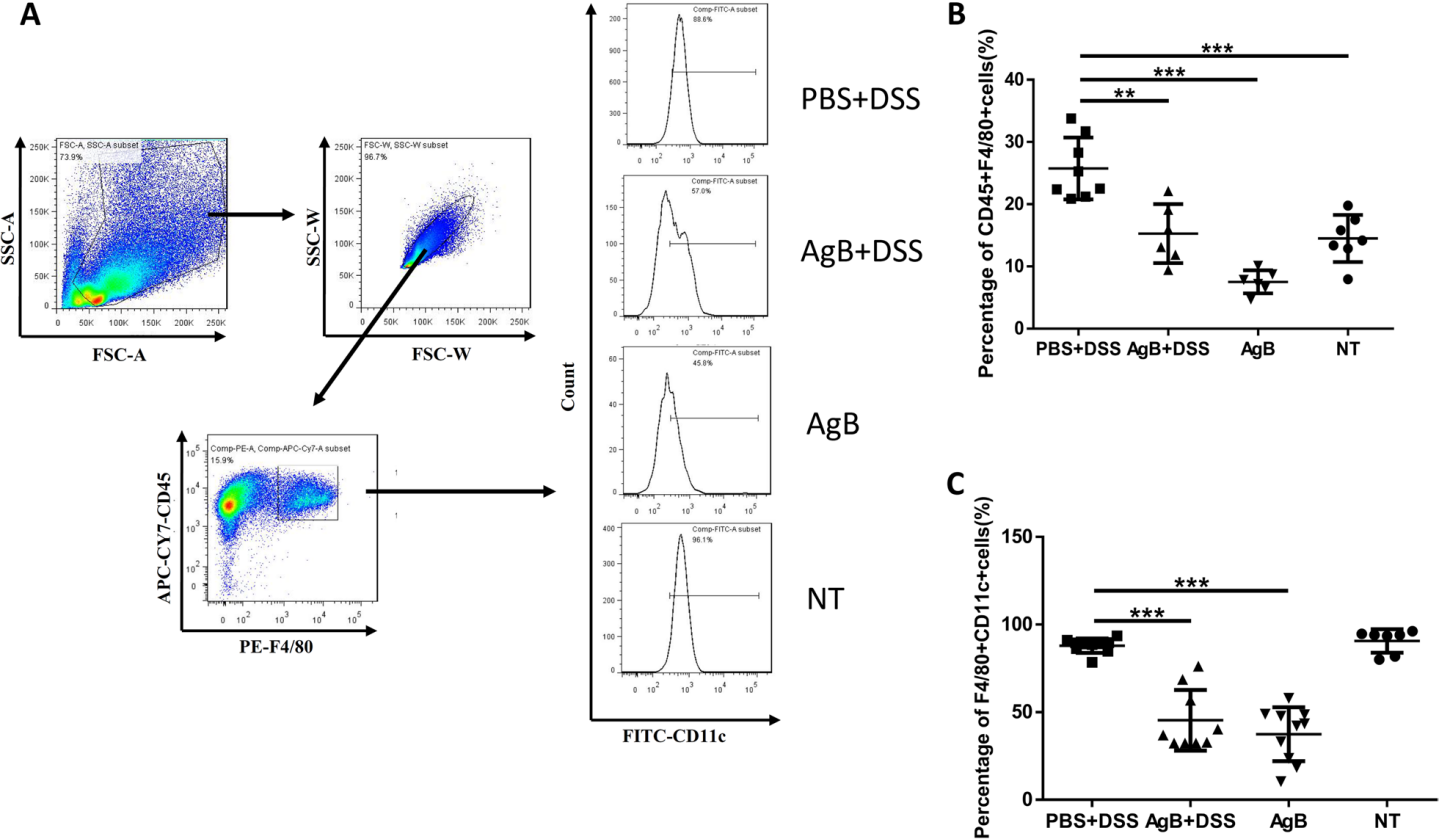
Cytoflow cytometric analysis of cells isolated from the peritoneal cavity of mice injected with AgB and challenged with DSS. **A** Cells were first gated on size and singularity followed by DAPI exclusion to identify live cells for further analysis. Live cells were gated on CD45 and F4/80 double expression to identify macrophages. Then, FITC-labelled CD11 expression was identified in M1 cells. **B** The frequency of macrophage marker CD45 and F4/80 double-positive (25.76 ± 4.97) was higher in the PBS+DSS group than in the other groups (*n* = 6–8). Bars represent the mean ± SD. **C** The proportion of type 1 macrophages (F4/80^+^CD11c^+^) was significantly decreased in the AgB group (37.46 ± 15.41) and AgB+DSS group (45.4 ± 17.30) compared with the PBS+DSS (87.99 ± 4.13) and NT groups (90.63 ± 6.68). Bars represent the mean ± SD for 7–10 mice per group. Comparison between groups was performed using one-way analysis of variance (ANOVA) with Bonferroni multiple comparison post-test for statistical analysis (the experiment was repeated independently,   ∗ *P* < 0.01, ∗ ∗ ∗ *P* < 0.005)

To analyse the effect of AgB on macrophage differentiation in vivo, we inoculated the protein into the peritoneal cavity of mice and then challenged the animals with DSS. Flow cytometry analysis showed that the frequency of macrophages (CD45^+^ and F4/80^+^) was higher in the PBS+DSS mouse group than in both the AgB group and AgB+DSS group (Fig. 5A, B). Type 1 macrophages (F4/80^+^CD11c^+^) were significantly decreased in the AgB group and AgB+DSS group compared with the PBS+DSS and NT groups (*P* < 0.01) (Fig. 5C).

### AgB inoculation changed the immune status in the colon of mice with DSS-induced colitis

**Fig. 6:**
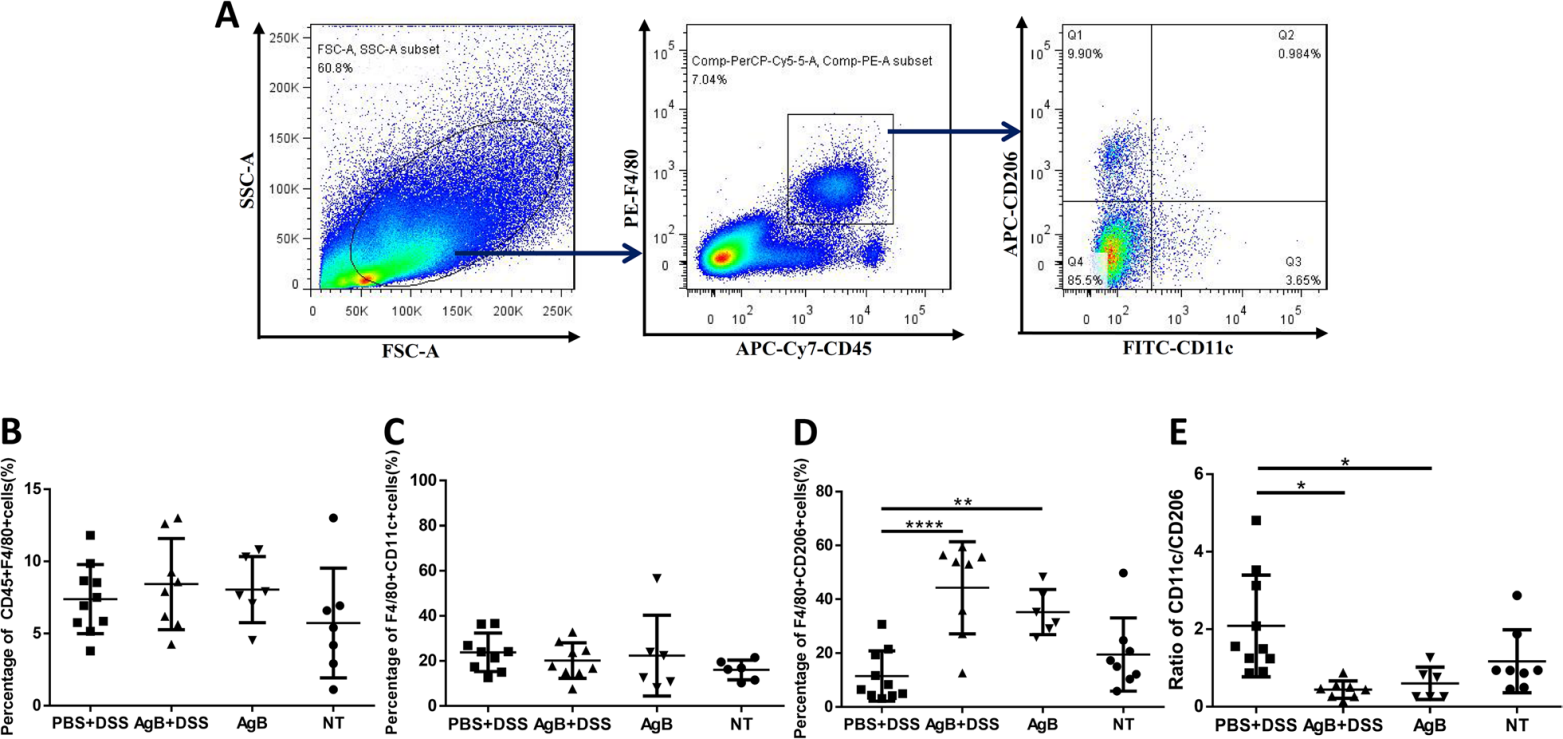
Injection with AgB altered the immune status of mice with DSS-induced colitis. LPMCs were collected from mouse intestines and tested by flow cytometry. Live cells were gated on CD45 and F4/80 double expression to identify macrophages; then, FITC-labelled CD11 expression was used to identify M1 cells, and APC-labelled CD206 expression was used to identify M2 cells. Comparison between groups was performed to determine the following: **A** There was no significant difference in the percentage of macrophages between the PBS+DSS group and other groups (*n* = 6–10). **B** The percentage of M1 cells between the groups also showed no significant difference (*n* = 6–10). **C** The percentage of M2 cells in the PBS+DSS group was reduced by 11.49 ± 2.95% compared with that in the AgB+DSS group (44.28 ± 6.04%, *n* = 6–10, *P* < 0.05). **D** The ratio of M1/M2 cells. Comparison between groups was performed using one-way analysis of variance (ANOVA) with Bonferroni multiple comparison post-test for statistical analysis (the experiment was repeated independently (*n* = 6–10). Bars represent the mean ± SD, ∗ *P* < 0.05, ∗ ∗ *P* < 0.01, ∗ ∗ ∗ ∗ *P* < 0.0001)

**Fig. 7:**
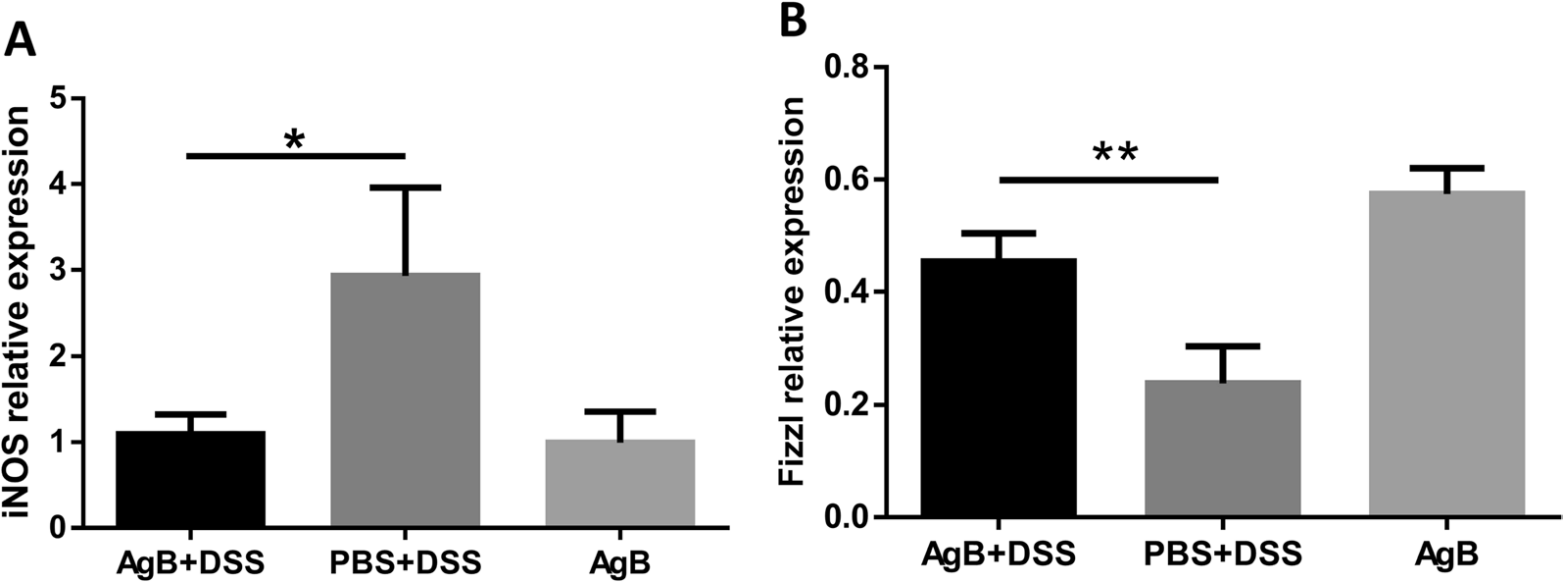
AgB intervention in mice altered the macrophage-specific gene expression of DSS-induced colitis. Expression of the transcripts was normalized, and fold change was calculated using the ΔΔCt method against mean control ΔCt; data are representative of three independent experiments. The relative expression of iNOS in the AgB+DSS group decreased significantly by 1.126 ± 0.294 compared with the PBS+DSS group (2.932 ± 1.026, *n* = 5–6, *P* < 0.05) (**A**). The relative expression of Fizz1 in the colon of the AgB+DSS group increased significantly (0.467 ± 0.063) compared with the PBS+DSS group (0.239 ± 0.066, *n* = 5, *P* < 0.05) (**B**)

DSS-induced acute colitis is characterized by an inflammatory reaction in the colon, with the production of a high level of inflammatory factors, including iNOS and tumour necrosis factor (TNF)-α. Intestinal macrophages play an important role in local immune responses. Colonic macrophages were isolated and evaluated by flow cytometry analysis. The flow cytometric gating strategy is presented in Fig. 6A. No significant difference was found in the proportion of macrophages, in particular type 1 macrophages, between the AgB+DSS and PBS+DSS groups (Fig. 6A, B). However, the proportion of F4/80^+^ and CD206^+^ M2 macrophages in the intestinal lamina propria of the AgB+DSS group was significantly increased compared with these macrophages in the PBS+DSS group (*P* < 0.005) (Fig. 6C). Notably, the ratio of CD11c^+^ to CD206^+^ cells differed significantly between the two groups (*P* < 0.05); AgB inoculation decreased the M1/M2 cell ratio in the colons of mice with DSS-induced colitis (Fig. 6D). qRT-PCR revealed that AgB significantly decreased iNOS expression (1.126 ± 0.294 and 2.932 ± 1.026, *P* < 0.05) and increased Fizz1 expression in the IBD model (0.239 ± 0.066 and 0.467 ± 0.063, *P* < 0.05) (Fig. 7).

### AgB influenced the gut microbiota of mice with DSS-induced colitis

**Fig. 8:**
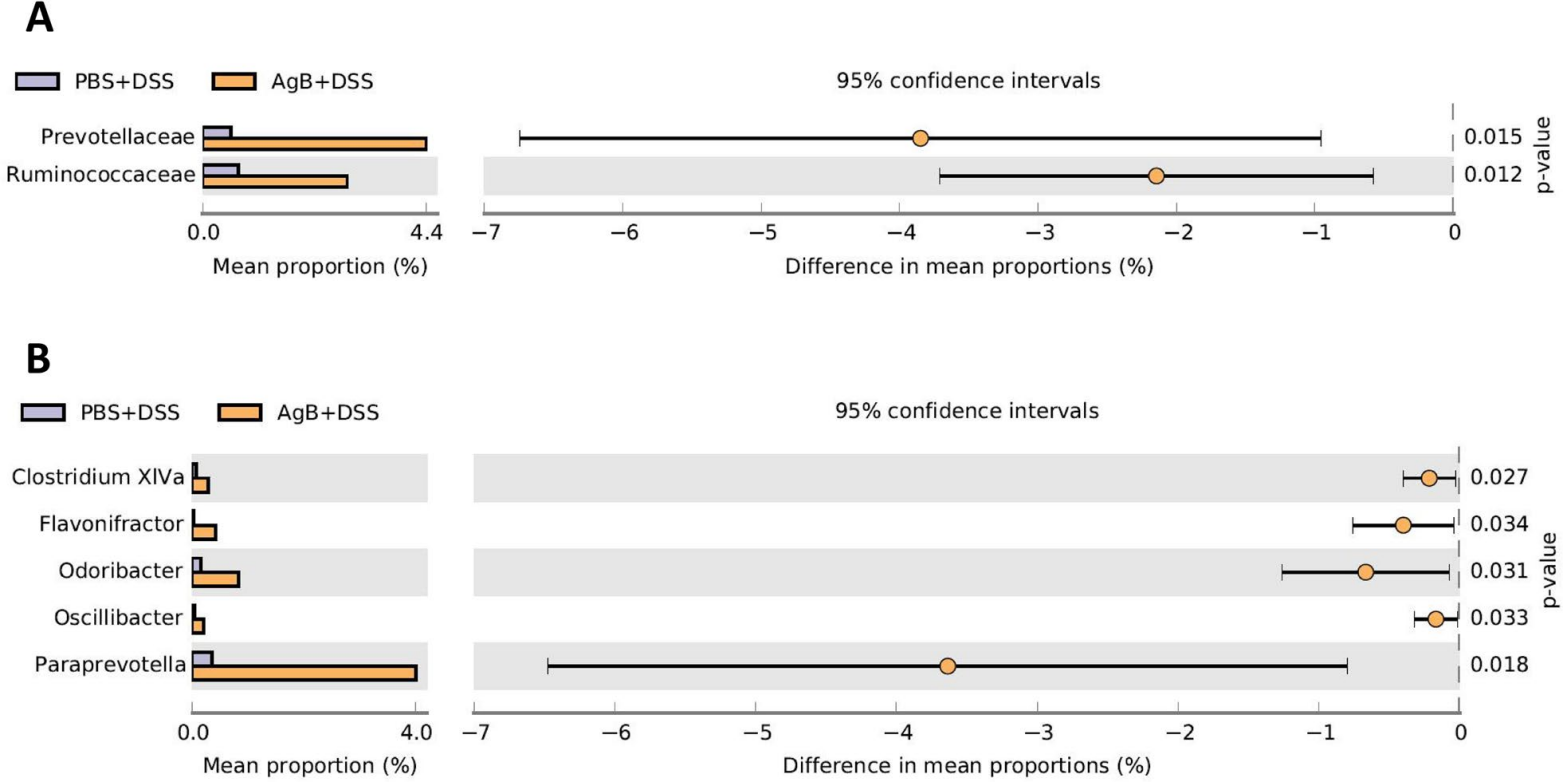
AgB influenced the gut microbiota of mice with DSS-induced colitis. Gut microbiota comparisons were made between the AgB+DSS and PBS+DSS groups at the family level (**A**) and genus level (**B**). There were five mice in each group. Gastrointestinal microbes were assessed using analysis of molecular variance (AMOVA) in Mothur

Crohn's disease and ulcerative colitis are heterogeneous diseases characterized by a dysregulated immune response to the normal gut flora, which is aggravated by environmental factors in genetically predisposed individuals. Linear discriminant analysis (LDA) effect size (LEfSe) analysis showed that the composition of the bacterial populations in the guts of *E. granulosus* s.s.-infected and uninfected mice was different. *Prevotellaceae* and *Ruminococcaceae* were increased at the family level in the AgB+DSS group compared with the DSS group (Fig. 8A). At the genus level, there were five genera (*Paraprevotella*, *Odoribacter*, *Clostridium* XlVa, *Oscillibacter*, and *Flavonifractor)* that were significantly different between the two groups (Fig. 8B).

### AgB ameliorated damage due to macrophages

**Fig. 9:**
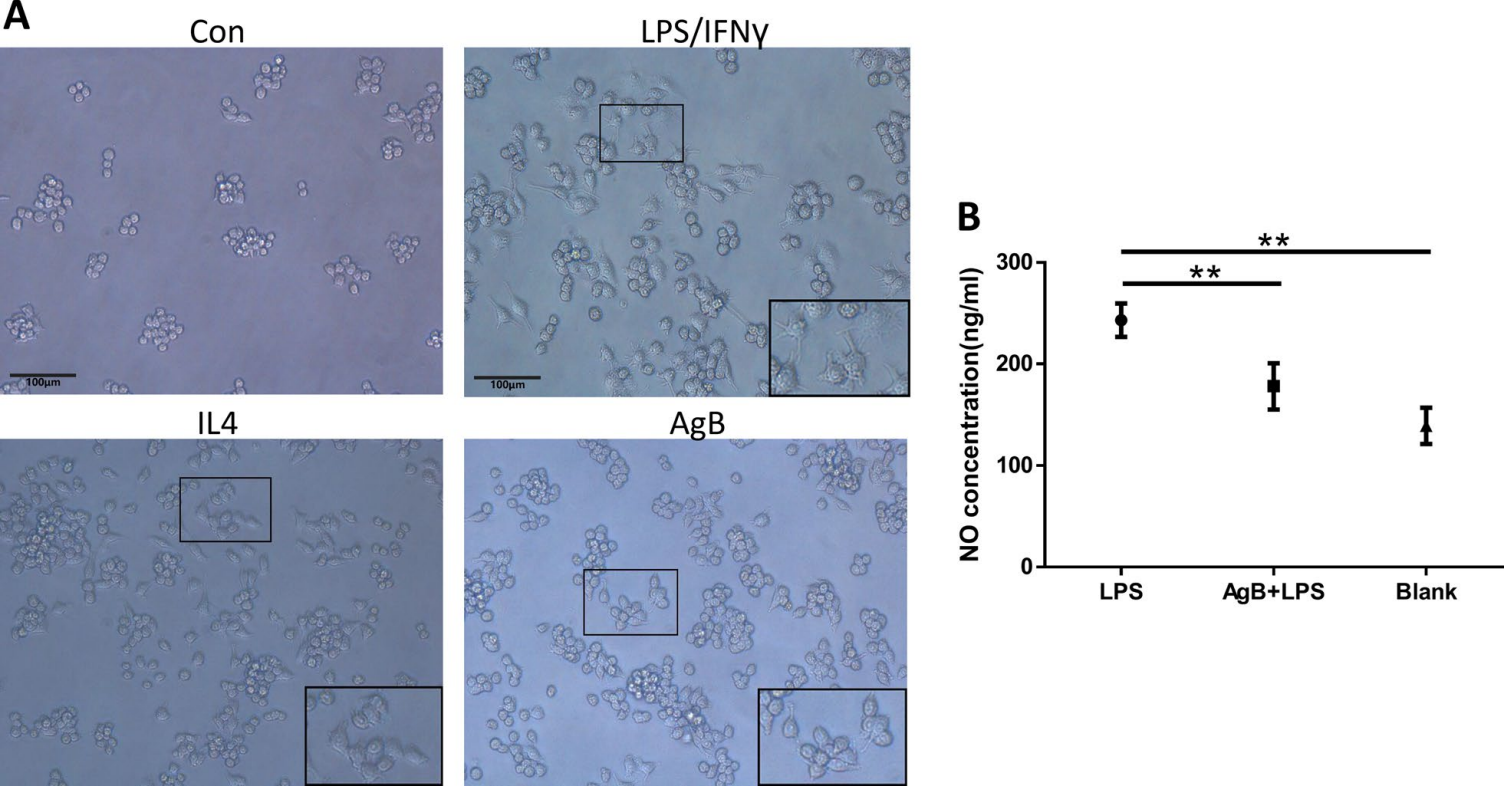
AgB intervention ameliorated the damage due to macrophages in vitro. **A** RAW264.7 cells were used to test the effect of AgB on macrophage differentiation (×200). **B** Macrophages were incubated with 1000 ng/ml AgB, and after 1 h, the cells were stimulated by the addition of LPS/IFN-γ. After 20 h, the amount of NO present in the cultured cell supernatant was measured using the nitric acid reduction method. In the LPS+AgB group, the level of NO was significantly decreased (178.11 ± 22.63) compared with the LPS group (243.18 ± 16.45). Comparison between the groups was performed using one-way ANOVA for statistical analysis (the experiment was repeated independently, *n* = 5, ∗ ∗ *P* < 0.01)

RAW264.7 cells were used to study the effects of B antigen (AgB) on the growth, polarization, and function of macrophages in vitro. After adhering to the wall of the culture plate, M1 macrophages were induced by adding LPS/IFN-γ, while M2 cells were induced by adding IL-4. AgB was added to RAW264.7 cells after 24 h in culture. The morphology of the cells in the LPS-stimulated group was altered, with most of the cells showing slender pseudopods; the cell bodies were narrow and long, indicating that the cells had differentiated towards M1 development. In contrast, the pseudopods of the cells in the IL-4 group were shorter, and the cell bodies were polygon-like in shape, indicating M2 status. Cells in the AgB group were mostly of round-like morphology and were likely in M0 status, similar to the PBS group (Fig. 9A).

LPS induces macrophages to polarize in the M1 direction with activation of iNOS and the production of NO. M1 macrophages induce inflammatory injury. To determine whether AgB could protect against M1-type inflammation caused by LPS in vitro, we firstly added AgB to the culture medium at a final concentration of 1 µg/ml 4 h after inoculation. Then, we added LPS/IFN-γ after 1 h and collected the cells at 20 h after the addition of LPS/IFN-γ. Compared with LPS-only-treated cells, AgB significantly reduced NO production following LPS stimulation (Fig. 9B), thereby reducing cell damage caused by LPS.

## Discussion

In this study, we determined that *E. granulosus* s.s. infection decreased the signs and symptoms of IBD in a murine model, thereby supporting the concept that helminth infection likely reduces the severity or consequence of autoimmune diseases.

Several studies have shown the ability of helminth parasites, including *Heligmosomoides polygyrus bakeri* [10], *Trichuris suis*, and *Schistosoma japonicum *[18], to attenuate intestinal inflammation in different animal models of colitis. However, the underlying mechanisms involved are poorly understood. We previously showed that *E. granulosus* s.s. infection impacted allergic asthma inflammatory responses through decreased eosinophil numbers in the airway and blood [11]. *Echinococcus granulosus* s.s. has been shown to regulate host immunity by biasing a Th1/Th2 response towards a chronic Th2 response [19], as the infection was found to induce a marked increase in the transcriptional levels of IFN-γ, IL-2, IL-4, IL-5, and IL-10 [11]. Mice infected with *E. granulosus* s.s. had significant levels of Th2-type immunoglobulins (IgG1, IgG2b, and IgE) against ECF antigens in their sera [11]. Similarly, a secondary infection with larval *Echinococcus multilocularis* infection reduced DSS-induced colitis in mice, which likely occurred through attenuation of the Th1/Th17 balance [20]. In another study, passive transfusion of M2 cells from mice infected with *Taenia crassiceps* into DSS-challenged mice significantly reduced IBD signs and symptoms [21]. These studies indicate that *E. granulosus* s.s. and other helminth parasites may share similar mechanisms by inducing M2 macrophages, which upregulate Th2 responses and reduce characteristic indicators of IBD. In fact, Th2 responses play a crucial role in the chronic phases of parasitic infection [22]. Th2-type cytokines, including IL-4, IL-10, and IL-13, may play a role in the reduction of IBD signs and symptoms.

Secreted antigens likely play an important role in the regulation of immune responses by parasitic helminths. Given that ECF is a major source of secreted *Echinococcus* antigens, we inoculated mice to determine whether this complex of native mixed antigens produced as *E. granulosus* s.s. infection had protective effects similar to the protective effects of *E. granulosus* s.s. infection on DSS-induced IBD, but we showed that this was not the case (Additional file 1). This may be due to the highly complex composition of ECF compounds.

In addition, a crude extract of the laminated cystic layer from *E. granulosus* s.s. has been shown to attenuate mucosal intestinal damage and inflammatory responses induced by DSS in mice [23], indicating that cyst wall antigens may contain antigens that stimulate immune responses against IBD. Another study showed that the laminated layer of the *E. granulosus* s.s. cyst is composed of AgB [24].

*Echinococcus granulosus* s.s. larval cysts can survive in human organs for long periods, up to 53 years [25], and release a large number of circulating antigens in serum, up to 680 ng protein per millilitre [26]. As AgB is the most immunogenic and abundant secreted echinococcal protein present in *Echinococcus* ECF [27], we purified this protein from ECF and undertook a similar series of experiments. We pretreated mice with AgB and then challenged these animals with DSS. The results showed a significant reduction in clinical symptoms induced by DSS. We thus consider that AgB may affect the polarization of macrophages and bias the host immune response to a Th2-predominant profile, thereby reducing IBD symptoms.

In this study, we showed that AgB decreased the number of type 1 macrophages (F4/80^+^ and CD11c^+^) in the peritoneal cavity of mice and increased F4/80^+^ and CD206^+^ M2 macrophage numbers in intestinal lamina propria cells. AgB pre-intervention decreased iNOS levels (*P* < 0.05) and increased Fizz1 levels (*P* < 0.05) (Fig. 7) in the IBD mouse model. Given that M2 macrophages play an important role in downregulation of IBD [28], AgB may play a core role in regulating the M2/Th2 response in CE patients, which may impact the IBD condition. Peritoneal macrophages play an important role in the control of inflammation and defence against abdominal infection [29].

AgB likely affects/regulates the host intestinal flora or microbiome, which may explain why *E. granulosus* s.s. reduces the inflammatory response in IBD and why AgB may be a reagent for prevention and protection against IBD, asthma, and other autoimmune diseases. The normal balance of the intestinal flora protects the intestinal mucosal barrier, so the disturbance of the micro-ecological balance of the intestinal microflora is a key factor in the development of IBD. Gut microbiota analysis indicated that AgB altered the number of *Prevotellaceae* and *Ruminococcaceae* in the gut of mice induced by DSS. Notably, this variation correlated strongly with disease status; i.e., inflammation had a significant impact on the microbiota composition [30]. Given that AgB was introduced by i.p. injection, alteration of the gastrointestinal microbiota may be an indirect function through the effects on the system immune responses. The loss of beneficial microbiota in the gut potentially contributes to chronic inflammatory diseases [31]. Additionally, helminths and the gut microbiota have co-evolved within their mammalian hosts [32], but the mechanisms of these interactions and the consequence of decreased exposure to intestinal helminths remain unclear. Helminths can reduce intestinal inflammatory responses by promoting the expansion of protective bacterial communities that inhibit proinflammatory bacterial taxa [33].

The intestinal bacteria of IBD patients are different from the intestinal bacteria of healthy persons, but the relationship between the characteristics of the flora and immunity in the intestine is unclear. IBD has been shown to be associated with *Parabacteroides* (vc23)[34]. We found a significant negative association between the numbers of *Parabacteroides* and the percentage of CD206^+^ cells in the colon, which were increased, likely through increased numbers of *Lachnospiraceae* (vc39) and *Parabacteroides* (*R* = 0.6). AgB increased CD206^+^ cells, likely by decreasing *Parabacteroides*, and reduced IBD signs and symptoms.

We showed that AgB inhibited the differentiation of macrophages to M1 macrophages. When cultured M0 cells were blocked with AgB and then stimulated with LPS, we found that far fewer M0 cells were differentiated into M1 cells, with more M0 cells developing to M2 cells. Meanwhile, the concentration of NO in the culture supernatant was lower than LPS-only-stimulated cells. We showed that AgB inhibited the secretion of NO by macrophages cultured with LPS, indicating that *E. granulosus* AgB may regulate macrophage differentiation to M2, which may be beneficial for treating and preventing IBD.

## Supplementary Information

**Additional file 1: Figure S1.** ECF showed no effect on mice with DSS-induced colitis. ECF was intraperitoneally injected into mice, which were then administered DSS for 7 days. The differences in parameters between the DSS and ECF-DSS groups are shown: (A) body weight change, (B) DAI score, and (C) colon length (8–10 mice per group). The experiment was repeated independently.

## Data Availability

The data generated or analysed during this study are included in this published article. Other datasets are available from the corresponding author on reasonable request.
