# Supplementary figures and images for: Echinococcus granulosus sensu stricto and antigen B may decrease inflammatory bowel disease through regulation of M1/2 polarization
Source: Parasit Vectors. 2022 Oct 27;15:391. doi: 10.1186/s13071-022-05498-y (PMC9608937; doi:10.1186/s13071-022-05498-y)

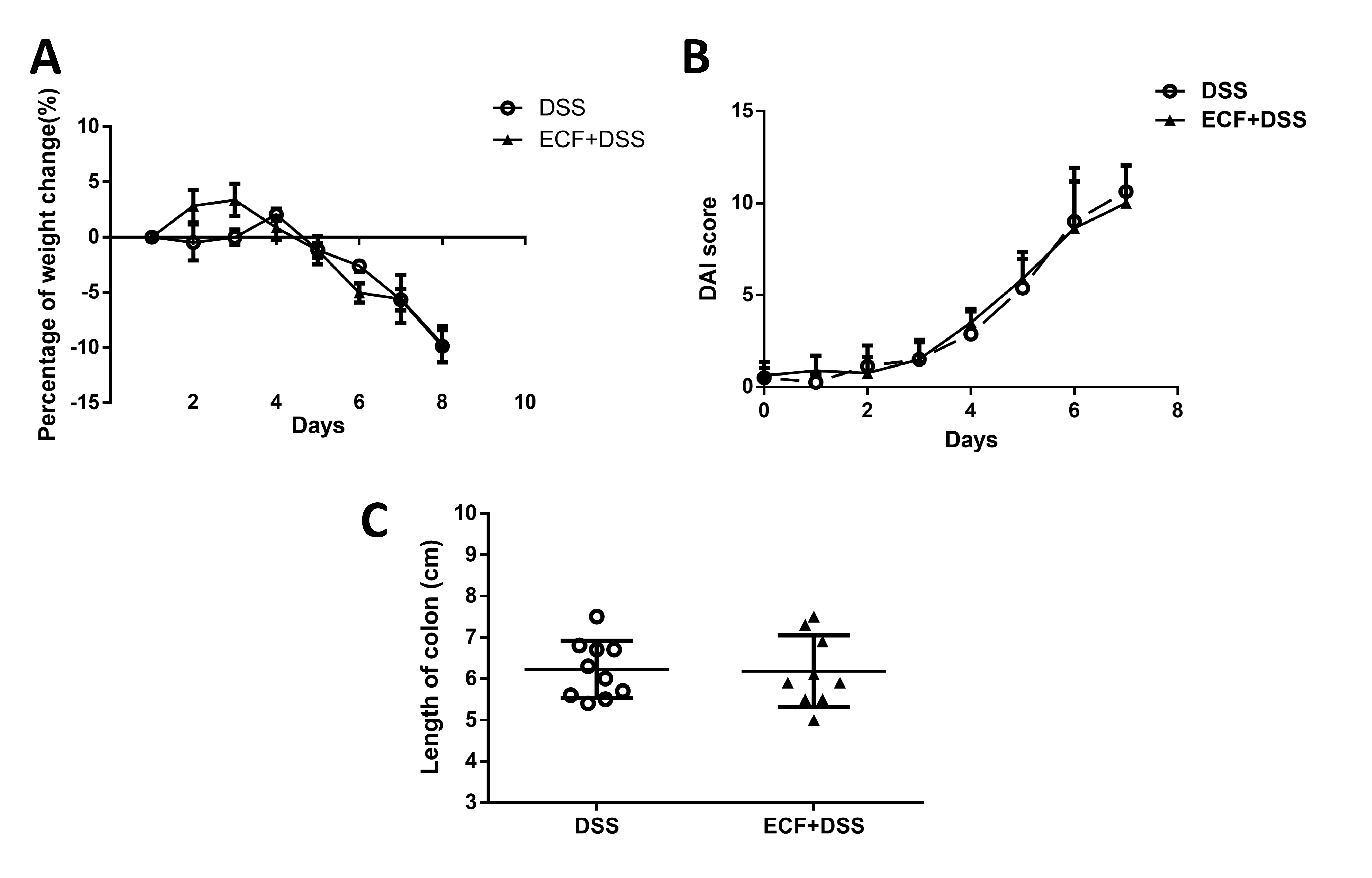

Supplement: Supplementary file 1 — Additional file 1: Figure S1. ECF showed no effect on mice with DSS-induced colitis. ECF was intraperitoneally injected into mice, which were then administered DSS for 7 days. The differences in parameters between the DSS and ECF-DSS groups are shown: (A) body weight change, (B) DAI score, and (C) colon length (8–10 mice per group). The experiment was repeated independently. [file 13071_2022_5498_MOESM1_ESM.tif]
